# Supplementary material for: Complex‐centric proteome profiling by SEC‐SWATH‐MS
Source: Mol Syst Biol. 2019 Jan 14;15(1):e8438. doi: 10.15252/msb.20188438 (PMC6346213; doi:10.15252/msb.20188438)
Supplement: Supplementary file 7 — Dataset EV6 [file MSB-15-e8438-s007.zip › feature_plots_bioplex/Q15397.pdf]

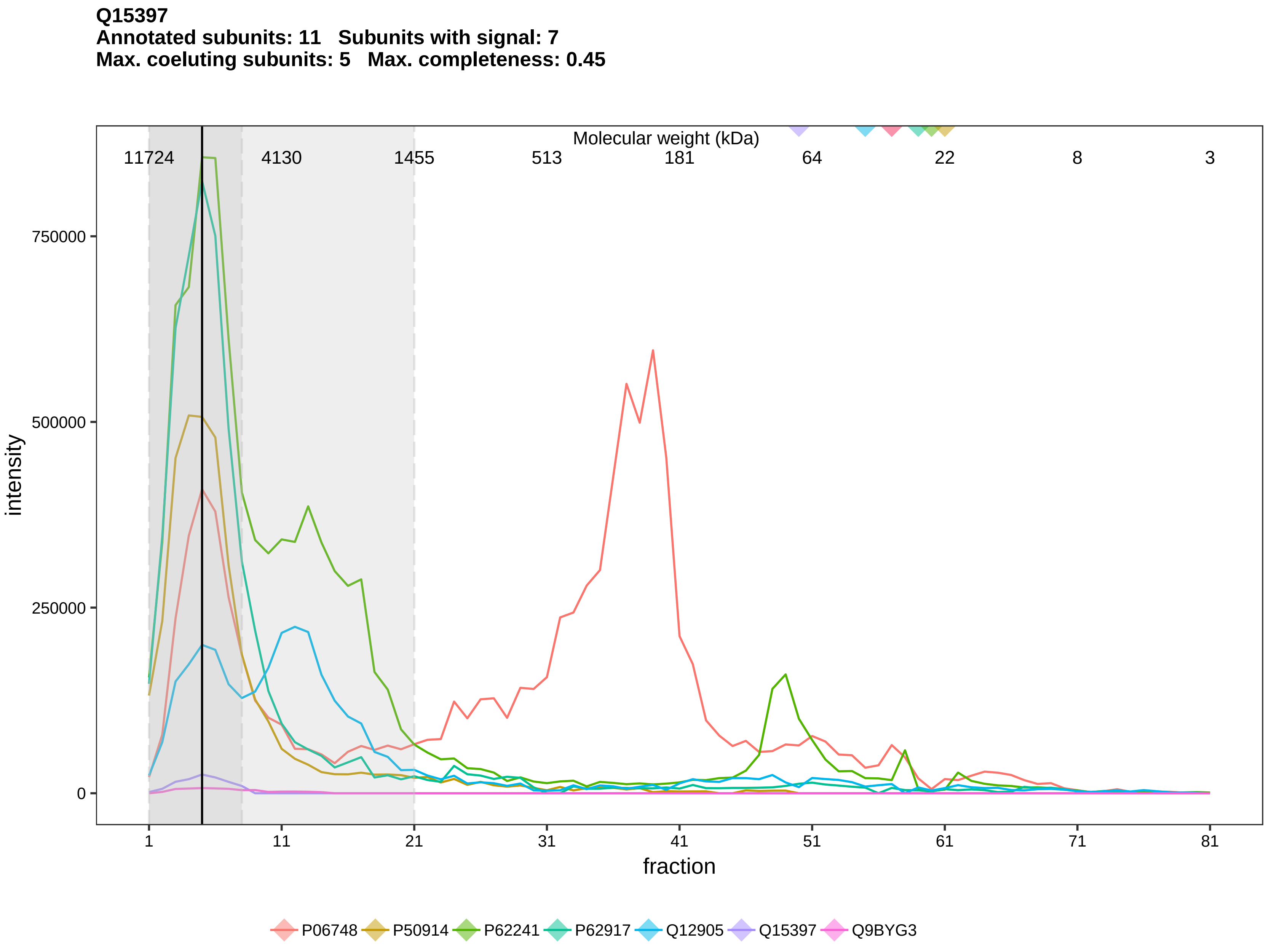

Q15397
Annotated subunits: 11 Subunits with signal: 7
Max. coeluting subunits: 5 Max. completeness: 0.45
Molecular weight (kDa)
11724
4130
1455
513
181
64
22
8
3
intensity
750000
500000
250000
0
fraction
1
11
21
31
41
51
61
71
81
P06748
P50914
P62241
P62917
Q12905
Q15397
Q9BYG3
